# ViromeXplore: integrative workflows for complete and reproducible virome characterization

**DOI:** 10.1093/bib/bbaf638

**Published:** 2025-12-05

**Authors:** Rodrigo Hernández-Velázquez, Michal Ziemski, Nicholas A Bokulich

**Affiliations:** Department of Health Sciences and Technology, ETH Zurich, Rämistrasse 101, 8092 Zurich, Switzerland; Department of Health Sciences and Technology, ETH Zurich, Rämistrasse 101, 8092 Zurich, Switzerland; Department of Health Sciences and Technology, ETH Zurich, Rämistrasse 101, 8092 Zurich, Switzerland

**Keywords:** viromics, metagenomics, workflow, Nextflow, virus

## Abstract

Viruses play a crucial role in shaping microbial communities and global biogeochemical cycles, yet their vast genetic diversity remains underexplored. Next-generation sequencing technologies allow untargeted profiling of metagenomes from viral communities (viromes). However, existing workflows often lack modularity, flexibility, and seamless integration with other microbiome analysis platforms. Here, we introduce “ViromeXplore,” a set of modular Nextflow workflows designed for efficient virome analysis. ViromeXplore incorporates state-of-the-art tools for contamination estimation, viral sequence identification, taxonomic assignment, functional annotation, and host prediction while optimizing computational resources. The workflows are containerized using Docker and Singularity, ensuring reproducibility and ease of deployment. Additionally, ViromeXplore offers optional integration with QIIME 2 and MOSHPIT, facilitating provenance tracking and interoperability with microbiome bioinformatics pipelines. By providing a scalable, user-friendly, and computationally efficient framework, ViromeXplore enhances viral metagenomic analysis and contributes to a deeper understanding of viral ecology. ViromeXplore is freely available at https://github.com/rhernandvel/ViromeXplore.

## Introduction

Viruses are the most abundant biological entities on the planet, outnumbering cellular organisms while providing important roles in regulating host-associated microbiomes [1, 2], global biogeochemical cycles [3], and microbial successions in diverse ecosystems. Viruses harbor a very high degree of genetic diversity, and a large proportion of these genes are of unknown function, further highlighting the need to characterize viruses and viromes [4].

Viromics has emerged over the last decades as one approach to study the vast diversity of viruses in different environments by leveraging next-generation DNA sequencing technologies for untargeted profiling of viral metagenomes. This can be accomplished via enrichment of viral particles in a sample [5] or by increasing the sequencing effort in a whole metagenome sequencing survey to adequately cover the viral genetic material (alongside sequencing of the more abundant bacterial or other nonviral DNA).

Mirroring advances in bacterial metagenomics, standard tools tailored for the specialized needs of virome analysis have been developed for routine operations such as genome assembly [6], viral sequence detection [7, 8], and taxonomic assignment [9]. With the emergence of new tools and more complex downstream analyses, workflows were developed initially for the study of bacteriophages, to compare different tools for bacteriophage identification [10], and more recently for all types of viruses [11, 12]. These recently developed workflows include basic functionality for the discovery of viral sequences, completeness estimation, taxonomic annotation, and gene prediction, among others [11, 12].

Nextflow is a workflow management system that is particularly relevant for high-performance computational (HPC) environments and allows for reproducible analysis and handling of containerized computation [13], making it the ideal framework for bioinformatic and virome workflow creation. Nextflow has also been used to generate multiple bioinformatics workflows given its scalability that allows local and HPC execution, parallelism to perform analyses on large datasets, modular development of pipelines, built-in support for containers, and resumability that allows continuing from the last successful step when the workflow is interrupted. These aspects are not available in Python wrappers for virome analysis, which, despite including relevant functionality, often require multiple steps for installation, large databases to be downloaded [11], and multiple modules that need to be run consecutively [14]. Other legacy pipelines like VIP and the actively maintained V-pipe have focused on the detection of viruses of relevance to public health and pathogens [15, 16], while others like TRACESPipe focus on the analysis of viral and human-host genomes at the multi-organ level [17]. These approaches are of high relevance for specific use cases, but this also limits their applicability to broader datasets and inputs.

Here, we introduce ViromeXplore, a set of Nextflow workflows that can be used with Docker or Singularity containers. These workflows were designed to provide flexible, modular, reproducible analyses that integrate the most recent and computationally efficient tools in the viromics field and are interoperable with other bioinformatics platforms for downstream analysis and visualization, such as QIIME 2 and MOSHPIT [18–20]. ViromeXplore complements existing approaches by providing new functionality such as viral genome elongation and network and phylogeny-based host prediction. Furthermore, it incorporates new tools such as ViromeQC [21] for contamination assessment or geNomad for taxonomy detection [22]. It also employs specialized curated databases for viruses and their corresponding hosts [23]. ViromeXplore provides a framework that can be applied to viromes, metagenomes, or viral genomes. It is user-friendly and can be configured according to the requirements of the user while considerably simplifying installation. ViromeXplore optionally allows running selected workflows using the popular QIIME 2 microbiome bioinformatics framework, further facilitating reproducibility and interoperability with downstream analysis tools.

## Materials and methods

All ViromeXplore workflows (available at https://github.com/rhernandvel/ViromeXplore) were implemented in Nextflow 22.04.4 [13] making use of the DSL2 syntax. Tools in these pipelines are installed using separate containers (Docker and Singularity) that allow reproducibility and facilitate maintenance of the workflows (https://github.com/rhernandvel/ViromeXplore). Parameters can be further adjusted, and custom databases can be specified according to the users’ needs (https://viromexplore.readthedocs.io/).

### Contamination estimation and read classification

ViromeQC (https://github.com/SegataLab/viromeqc) is used in the pipeline to quantify nonviral contamination. This tool screens for microbial marker genes (16S/18S rRNA genes, 23S/28S rRNA genes, and a set of 31 universal bacterial genes) present in the virome sequences and provides an enrichment score [21]. This program takes into account the source of the sample, and the default for this pipeline is environmental origin, although it can be switched to human origin. The reads are also taxonomically classified as part of this pipeline by using Kaiju, which classifies reads based on exact matches at the protein level [24]. The default database is specific for viral sequences (kaiju_db_viruses). In addition to the classification table output provided by Kaiju containing the read classification, a Krona plot [25] is obtained per sample, which allows the user to explore the taxonomic composition results interactively.

### Virus assembly

The virus assembly workflow takes short raw reads as input in FASTQ format as they are obtained from the sequencing platform of choice. These reads are subject to trimming and filtering using fastp [26] with the default settings, except for the quality value for base pairs that was set to a Phred score of 30, which can be modified by the user. The quality of the reads can be explored interactively through an HTML file generated for each sample. MEGAHIT [27] was implemented for the read assembly that takes place in any metagenome or virome study. The default parameter for the kmers for this workflow is set to: 21,35,49,63,77,91,105,119,127.

### Viral sequence identification

VirSorter2 [7] is used for viral sequence identification, setting the —keep-original-seq option to skip trimming of the sequences for downstream analysis. Sequences <1500 bp are removed, and the classification is set for the dsDNAphage, Nucleocytoplasmic large DNA viruses (NCLDV), RNA, ssDNA, and lavidaviridae groups by default. The default score cutoff (0.5) for this value was kept. CheckV end_to_end is used for quality control and completeness estimation of the preselected viral sequences [28]. High-quality sequences that correspond to viral genomes are then clustered with the Cluster Database at High Identity with Tolerance (CD-HIT) program, considering a sequence threshold of 95% for the global sequence identity for both strands of each sequence [29]. The default clustering coefficient was set to 0.95, considering that this Average Nucleotide Identity (ANI) threshold is frequently used to obtain viral operational taxonomic units (vOTUs) [30, 31], and it has been shown to represent genotypic cluster for double stranded DNA (dsDNA) populations [32].

### Taxonomic assignment and functional annotation

The taxonomy and annotation pipeline takes as an input a FASTA file containing viral contigs or genomes that have previously been assembled or fetched from a genome repository. The taxonomy is initially obtained through geNomad using the end-to-end command [22]. This software also performs a classification of the contigs or genomes and distinguishes between viral and plasmid sequences. Then, viral nucleotide sequences identified previously are globally aligned against the Virus-Host Database reference [23] using VSEARCH [33] with a global clustering value of 0.95. Finally, predicted genes are annotated using eggNOG-Mapper v2 [34] using the default Diamond mode [35] and EggNOG database [36].

### Viral genome abundance estimation and elongation

Viral genome abundance estimation is performed using Bowtie2 [37] to map the reads to the contigs and SAMtools [38] to sort them; then, the abundance is calculated using the jgi_summarize_bam_contig_depths from MetaBAT2 [39]. To improve the completeness and contiguity of the viral genomes, the tool COBRA (Contig Overlap Based Re-Assembly) is used, taking as input the mapping, coverages, and contig files generated previously [40].

### Network- and phylogeny-based host prediction

The network- and phylogeny-based host prediction uses the approach described by Kaneko *et al.* [41]. As an input, it requires a count matrix (feature table) that contains the relative abundances of both the viruses and hosts in each sample, a phylogeny for the viral sequences and a table containing the National Center for Biotechnology Information (NCBI) taxonomic lineage for the host operational taxonomic units. These count matrices are not generated by the ViromeXplore core workflows (as host abundance must be measured separately), but these matrices can be generated separately, e.g. via QIIME 2 and MOSHPIT.

FlashWeave is used to construct co-occurrence networks using the heterogeneous and sensitive modes [42]. The network connections are then used as an input, along with the viral phylogenetic tree and the eukaryotic lineage (NCBI taxonomic terms) for the Taxon Interaction Mapper (TIM) tool [41]. The results from this tool can be visualized interactively through the Interactive Tree of Life (iTOL) tool [43].

### QIIME 2 workflows

QIIME 2 is a software platform for reproducible microbiome multi-omics data analysis, and it includes many tools and visualization methods, making it a comprehensive and popular environment for analysis [18]. Furthermore, QIIME 2 tracks analytical steps via its unique integrated data provenance functionality, enabling full transparency and reproducibility, e.g. via provenance replay [19] that allows users to generate executable scripts to repeat prior analyses. For this reason, we provide a version of the workflows that incorporates the actions included in the q2-viromics plugin (https://github.com/bokulich-lab/q2-viromics) and produces QIIME 2 artifacts. This accessory plugin of the MOSHPIT toolkit includes multiple actions related to viral sequence analysis that are further complemented in these workflows by other metagenomics actions [20]. A separate version of the workflows is available that does not incorporate QIIME 2, so that users have the option to run the full workflow independent of QIIME 2 (e.g. to skip provenance tracking functionality).

### Analysis of human viromes

To test and showcase the functionality of ViromeXplore, we reanalyze data from a study of viromes from persons in Cameroon living in close proximity to bats [44]. From this study, the sequencing reads from 65 participants in the 20–59 age group were selected, which corresponded to a dataset of 17 metagenomes. This study was selected due to the extraction of both RNA and DNA, as well as the varying sequencing effort per sample, ranging from 3.8 to 26.6 million reads.

The FASTA sequences and metadata were downloaded using the QIIME 2 plugin q2-fondue [45]. For these samples, the workflows (1) Virus assembly, (2) Contamination estimation and read classification, (3) Viral sequence identification, (4) Viral genome abundance estimation and elongation, and (5) Taxonomic assignment and functional annotation were consecutively used.

### Recovery of viral genomes from metagenomes

To demonstrate the feasibility of recovering viral genomes directly from metagenomes, as opposed to exclusively from viromes, metagenomic reads from a fermented baked milk product Ryazhenka sample were downloaded using q2-fondue [45]. To analyze this dataset, the following workflows were applied sequentially: (1) Virus assembly, (5) Taxonomic assignment and functional annotation, (4) Viral genome abundance estimation and elongation and (3) Viral sequence identification. In this sample, only the single complete genome that met high-quality standards (>90% completeness) according to CheckV was retained for further analysis.

A comparative genomics analysis was performed outside of ViromeXplore to demonstrate how outputs are directly interoperable with downstream tools. First, to explore genomic similarity, a complete Streptococcus phage genome obtained from the Ryazhenka sample was used as query for a BLASTn search against the NCBI nucleotide (nt) database [46]. The top three hits were selected for comparison, along with the reference genome of Streptococcus phage DT1 (accession number: NC_002072.2). Then, gene prediction was performed on the complete genome using Prokka [47], and synteny analysis was carried out by performing pairwise alignments of protein-coding regions in all genomes using BLASTp [46]. The results were filtered to include only alignments with ≥30% sequence identity and aligned regions of at least 100 amino acids in length. A synteny plot was then generated using the pyGenomeViz library (https://github.com/moshi4/pyGenomeViz).

### Classification, assembly, and viral detection for a synthetic viral community

In order to benchmark ViromeXplore, a synthetic viral dataset was generated from 15 viral genomes that have been well characterized and infect diverse hosts including humans. These viruses were selected based on their varying genome sizes and types of genetic material, ensuring representation of both DNA and RNA viruses (Supplementary File 1). Reads for the synthetic viral community were generated using the ART program [48]. This was achieved by generating reads for the genomes with relative abundances drawn from a log-normal distribution. The ViromeXplore workflows: Contamination estimation and read classification, Virus assembly, Viral sequence identification, Viral genome abundance estimation and elongation, and Taxonomic assignment and functional annotation were consecutively used to analyze the synthetic viral community. MetaQUAST 5.3.0 was used to compare the detected viral sequences against the reference genomes [49].

## Results

### ViromeXplore implementation

**Figure 1:**
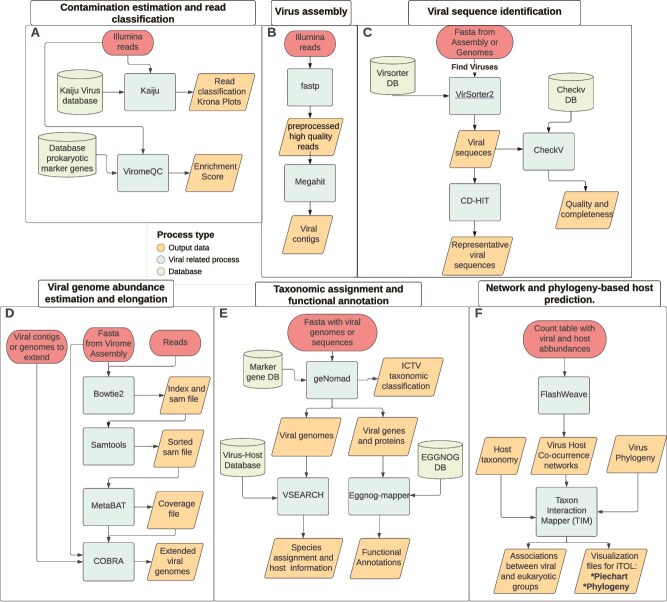
Workflows included in ViromeXplore: (A) contamination estimation and read classification, (B) virus assembly, (C) viral sequence identification, (D) viral genome abundance estimation and elongation, (E) taxonomic assignment and functional annotation, and (F) network- and phylogeny-based host prediction.

The presented workflows are complimentary and expand the functionality of several approaches like ViRify [11] and ViromeflowX [12] (Fig. 1). A summary of the tools incorporated into ViromeXplore and a comparison with these workflows is visualized in Table 1. ViromeXplore runs in Nextflow and utilizes Docker or Singularity containers. It is compatible with HPC systems using Slurm or LSF. ViromeXplore allows to visualize many of the outputs using Krona plots [25], quality control statistics through fastp [26], and phylogeny visualization through an iTOL readable file [25]. The underlying tools incorporated in ViromeXplore were selected to include the most efficient and adequate approach for each process based on independent benchmarks when available.

**Table 1:** Functionality comparison between ViromeXplore and the two most comprehensive workflows for virome analysis: Virify and ViromeflowX.

	**Virify**	**ViromeFlowX**	**ViromeXplore**	**ViromeXplore advantages and intercompatibility**
**Contamination estimation and read classification**		KRAKEN 2 [60]	ViromeQC [21] KAIJU [24] Krona [25]	ViromeXplore enables an estimation of the contamination in the virome and a lightweight, computationally efficient read classification.
**Virus assembly**		Trimmomatic [61] Bowtie2 [37] metaSPAdes [62]	Fastp [26] MEGAHIT [27]	ViromeXplore provides a more efficient approach by using fastp and MEGAHIT, which is the fastest assembler.
**Viral sequence identification**	Virsorter2 VirFinder PPR-Meta [63] CheckV	Virsorter2 VirFinder [8] CheckV CD-HIT	Virsorter2 [7] CheckV [28] CD-HIT [29]	ViromeXplore offers a lightweight workflow for the identification of high-quality viral sequences.
**Viral genome abundance estimation and elongation**		Bowtie2 CoverM [64] BEDtools [65]	Bowtie2 [37] SAMtools [38] MetaBAT2 [39] COBRA [40]	ViromeXplore allows to obtain longer and more complete viral genomes through COBRA.
**Taxonomic assignment and functional annotation**	ViPhOGs [11] HMMER [66]	RefSeq [67] Pfam [68] Demovir [69] Diamond [35] UniRef [70]	geNomad [22] VSEARCH [33] Virus-Host DB [23] eggNOG-mapper v2 [34] EggNOG [36]	ViromeXplore uses genomad and its comprehensive viral marker gene database. It uses Virus-Host DB, allowing to obtain relevant host information. Fast annotation is enabled through eggNOG-mapper.
**Network- and phylogeny-based host prediction**			FlashWeave [42] TIM [41]	ViromeXplore allows to predict viral hosts based on co-occurrence and viral phylogeny.

### Tool selection criteria

After an extensive literature search, the following criteria were used to select the most efficient and adequate tools based on performance and applicability to virome datasets:

- To our knowledge, ViromeQC is to date the only tool that provides an enrichment score for contamination in viromes [21].
- Kaiju has been established as the best protein classifier according to speed and memory utilization while also showing the closest results to the real species abundance [50].
- Fastp was found to be two to five times faster than other FASTQ preprocessing tools [26].
- MEGAHIT is regarded as the assembler with the best performance, especially for deeply sequenced samples [51]; furthermore, MEGAHIT has been used successfully in studies of diverse viromes in different ecosystems [1, 31, 52]. Other tools like METAVIRALSPADES are specific to obtain viral contigs from metagenomes [6]; this tool uses initially metaSPAdes, which requires considerably more resources than MEGAHIT [53].
- VirSorter2 was selected for viral sequence prediction, considering that a benchmark study showed it achieved a higher F1-score than competing tools when using a reference dataset [54]. Furthermore, VirSorter2 identifies diverse types of DNA and RNA viruses, expanding previous tools that focused exclusively on bacteriophages [55].
- CheckV is the golden standard tool for estimating genome completeness and contamination for single-contig viral genomes [28].
- CD-HIT [29] was selected to cluster the viral sequences in each virome and metagenome, given that approaches using average nucleotide identity like fastANI [56] are computationally very demanding for regular virome datasets.
- Read mapping with Bowtie2 and sorting with SAMtools [37, 38] has become a golden-standard practice in metagenomics, and the former program has been shown to perform well with default parameters in metagenomic analyses [57].
- MetaBAT2 is the binner that is most widely used in large-scale metagenomic studies and the one with the highest computational and memory efficiency [39, 58].
- Even though there are some tools to perform virus binning, COBRA outperforms them considerably reducing significantly the contamination and performing better in precision, recall, F1 score, specificity, and accuracy [40].
- One of the most recent tools to classify mobile genetic elements is geNomad, which uses a hybrid approach consisting of an alignment-free method (neural network) and gene-based taxonomic assignment. This approach allows the identification of viruses and plasmids with a higher performance compared to other tools [22].
- For global alignments, VSEARCH [33] offers a fast, open-source option for comparing entire sequences to classify and identify viral species accurately in viromes.
- For gene annotation, eggNOG-Mapper v2 is used, which is now optimized for large metagenomic data sets and has improved annotation rates by 608% on average, compared to the previous version, which was already considerably faster than other tools [34, 59].
- The Virus-Host DB contains the largest manually curated number of virus–host pairs with validated information from RefSeq, GenBank, UniProt, ViralZone, and additional information obtained through literature surveys [23].
- Flashweave is the fastest, most accurate, and computationally efficient tool to predict direct associations based on abundance matrices, outperforming other network inference methods [42].
- Taxon Interaction Mapper (TIM) combines the input of a co-occurrence network, a viral phylogeny, and host taxonomy to predict virus–host pairs [41].

### Full human virome analysis

**Figure 2:**
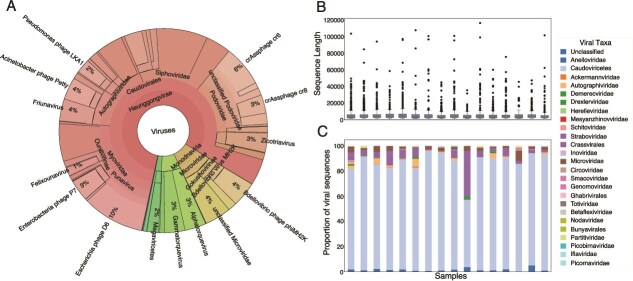
(A) Krona plot showing read classification for one of the viromes. (B) Viral genome size distribution per metagenome. (C) ICTV viral genome taxonomy per sample as determined by geNomad.

The utility of ViromeXplore for virome profiling was demonstrated using a dataset of human viromes from rural Cameroon. Within this cohort, a subset of individuals had a disrupted microbiome due to diarrheic outbreaks and had also been in close proximity to bats [44]. Thus, this dataset was selected due to the high relevance of viromics for the investigation of novel zoonotic infections. Figure 2A shows the read-based viral taxonomic composition for one of the samples displaying the dominance of Caudovirales (tailed bacteriophages) that account for 80% of the viral reads, followed by Monodnaviria (9%) and Anelloviridae (7%). Contigs were successfully assembled, and both DNA and RNA viral sequences were detected. The number of viral contigs obtained after running all the workflows was considerably lower compared to all the contigs in the assembly; this can be attributed to the presence of bacterial and metazoan contigs as reported in the original publication and by the clustering performed in the “Viral sequence identification” workflow. The extension of the viral sequences using the “Viral genome abundance estimation and elongation” workflow led to a per-sample extension between 1.07% and 4.3% of the total viral contigs. The average length of the viral genomes obtained after running the workflow is shown in Fig. 2B. This result shows the broad distribution of genome sizes that can be attributed to the viral diversity and differences in sizes within DNA viruses (mainly phages) and between RNA and DNA viruses.

When evaluating the viral sequences, the taxonomy assigned by geNomad showed that both DNA and RNA viral clades were successfully obtained using ViromeXplore. RNA Viral families mentioned in the original study were also reported including the Picornaviridae, Nodaviridae, Partitiviridae, Bunyaviridae, and Totiviridae. Noteworthy families like Picornaviridae are known to cause gastroenteritis and are thus of high relevance, given the background of the samples [71]. Meanwhile, other families like Iflaviridae and Betaflexiviridae that were not reported in the previous study were found with the present approach (Fig. 2C).

**Figure 3:**
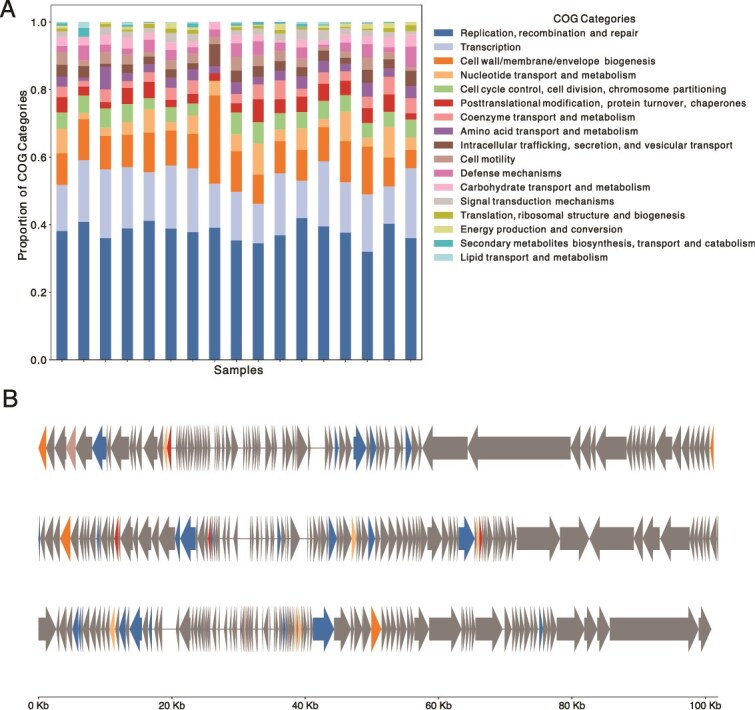
(A) COG category assignment of all the viral genes per metagenome. (B) Three largest Crassvirales genomes and marker genes assigned by geNomad.

From the functional annotations through eggnog-mapper, the Cluster of Orthologous Groups (COG) categories were extracted per gene and those with >10 occurrences were included in Fig. 3A. The most represented COG category corresponded to “Replication, recombination, and repair,” which is the most important biological function for the viral cycle; this category was followed by “Transcription” and “Cell wall/membrane/envelop biogenesis.” Finally, the three largest “Crassvirales” genomes were selected for visualization along with their marker genes; these sequences were larger than 100 kb and represent complete genomes shown in Fig. 3B.

### Streptococcus phage retrieved from fermented food metagenome

A second demonstration analysis was performed using metagenomic data from Ryazhenka (a fermented baked milk product), showing performance on mixed metagenomes (i.e. containing viral and nonviral reads). The metagenome was shown to have a large fraction of reads classified as viral and according to a Kraken 2 analysis, these reads mapped to the Streptococcus phage DT1 [60, 72]. The Streptococcus phage genome found had a total length of 35 170 bp and a total average depth of 15 447x, the highest of all viral sequences that was also 6.5 times higher than the next most abundant viral genome.

**Figure 4:**
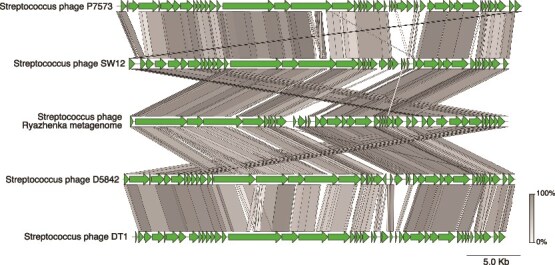
Streptococcus bacteriophage genome synteny. The streptococcus phage obtained from the Ryazhenka metagenome is shown in the middle; the gray scale corresponds to sequence similarity.

The synteny analysis revealed that the Streptococcus phage found in the Ryazhenka sample had a genomic rearrangement where several proteins related to the tail, capsid, and terminases were located in the opposite end of the genome (Fig. 4). This kind of mosaic genome structure has been described for other dairy *Streptococcus thermophilus* phages [73].

### Viral sequences detected in the synthetic viral community

In total, 20 million reads were generated with the abundances per viral genome described in the Supplementary file 2. Read level classification with Kaiju revealed that viruses composed 100% of the virome. Furthermore, the proportion for the different viruses was kept and showed a high level of correspondence to the proportion used to generate the synthetic dataset, both for high-abundance and low-abundance genomes. Such is the case for the *Acanthamoeba polyphaga* mimivirus that represented 26% of the community (26% of the reads classified as the same taxa by Kaiju) and lower-proportion genomes like the Zika virus, which comprised 3.6% of the reads (4% of the reads classified by Kaiju). A total of 21 long viral sequences were detected after running ViromeXplore for this simulated virome. Twelve sequences were classified as high-quality, while the rest were classified as genome-fragment according to CheckV. After running MetaQUAST, it was found that the viral sequences obtained with ViromeXplore covered 99.72% of the reference genomes and had 0.05 misassemblies per 100 kb. While almost all genomes were obtained as a single sequence and taxonomically classified, three were obtained as fragmented contigs. Two of these genomes were split into two contigs each (Salmonella phage P22 and Enterobacteria phage lambda), while the *A. polyphaga* mimivirus genome was fragmented into five sequences (ranging from 22 to 906 kilobase pairs). The remaining genomes, besides being represented by a single contig, had a percentage identity >99.4% and were fully covered in all their length.

## Discussion

ViromeXplore has a set of modular workflows that allow full virome exploration and that can be used in different orders and combinations to characterize viral sequences and address different biological questions. It contains functionality that is not present in other workflows such as a network- and phylogeny-based host prediction. This prediction is independent of virus–host databases, which makes it a complementary approach to workflows that infer virus–host pairs directly from sequence analysis. ViromeXplore also enables the use of the most recent tools like geNomad [22], which allows to simultaneously detect plasmid sequences that are mobile genetic elements that can be misidentified as viruses and bacteriophages; furthermore, it also outputs a taxonomic classification according to the International Committee on Taxonomy of Viruses (ICTV) [74].

Fast taxonomic profiling of sequence reads is an essential component to obtain an initial estimation of contamination after sequencing a virome. ViromeflowX uses the nucleotide-based tool Kraken2 [60] for taxonomic classification, while ViromeXplore uses Kaiju and its corresponding viral database [24]. Although read-based classifications can provide a higher taxonomic resolution for lower taxonomic levels, it is worth considering that viromes are composed of highly divergent sequences, most of which do not have closely related reference matches. Hence, a protein-based classification is desirable to preserve distant evolutionary relationships. A step that is often omitted is determining the contamination in a virome. This is a crucial given that the enrichment and purification of viral-like particles is imperfect and bacterial, archaeal, and fungal contamination is often present [21, 75]. To address this, we incorporated ViromeQC, which estimates prokaryotic and eukaryotic contamination using corresponding universal marker genes [21].

To our knowledge, ViromeXplore is currently the only workflow that incorporates the virus–host DB [23], which contains curated virus–host information and can be used to quickly detect well-characterized viruses, including human pathogens and viruses of interest to public health. To annotate the viral genes, eggNOG-mapper V2 is included in the “Taxonomic assignment and functional annotation” workflow. Besides its optimal performance when used with the eggNOG 5.0 database [36], it provides a context-rich report including predicted protein name; Kyoto Encyclopedia of Genes and Genomes (KEGG) pathways, modules, and orthologs; Gene Ontology labels; Enzyme Commission (EC) numbers, BiGG reactions; CAZy terms; COG functional categories; eggNOG OGs; and free text descriptions at all taxonomic levels [34].

Both workflows used for comparison do not include a step to bin viruses and only read mapping to the viral contigs is performed by ViromeflowX. ViromeXplore integrates a workflow that is suggested by the authors of COBRA, a tool that has been shown to be highly efficient in recovering novel phage species [40]. By combining sequence agnostic host prediction, complete and detailed taxonomic characterizations, and functional annotations, it is possible to generate hypotheses and infer ecological roles that viruses in different ecosystems play.

**Figure 5:**
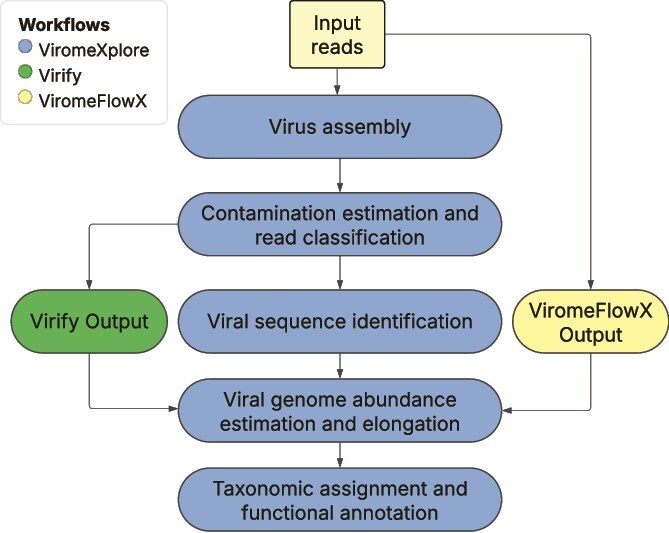
Integration of ViromeXplore workflows with other existing workflows for the characterization of viromes.

Pipelines contained in ViromeXplore such as the “Viral genome abundance estimation and elongation” and “Taxonomic assignment and functional annotation” can be run subsequently after other workflows like Virify, which predicts viral sequences using three tools to later combine the results [11]. In a similar way, ViromeflowX uses both Virsorter2 [7] and Virfinder [8] to later merge the results and can be complemented by the pipelines presented here (Fig. 5). An alternative to these approaches for viral sequence prediction is to use the lightweight “Viral sequence identification” workflow that only incorporates one tool and clusters the resulting sequences with CD-HIT [29]. This last clustering step is also conducted by ViromeflowX, and it allows to reduce redundancy and conserves computational resources [12].

The high modularity of the ViromeXplore pipelines allows the user to apply them for different tasks while maximizing compatibility with other tools and available resources. For example, computationally expensive workflows are provided as separate modules so that users can select the modules that best fit their resources and experimental needs. ViromeXplore saves computation time and storage space by only downloading the containers and databases of the workflows being used. The user can also provide custom databases that are compatible with the different tools and adjust relevant parameters for improved performance. Of note, the tools selected for the different pipelines offer the best tradeoff between efficiency and accuracy according to independent benchmarks. These tool comparisons, despite being conducted with limited parameters and databases, offer valuable insights that require periodic revision. Another key aspect for initial exploration of the data is visualization, which is interactively enabled in workflows and through helper scripts that will be further expanded in future releases.

Finally, a synthetic viral community spanning a broad range of viruses was generated and successfully analyzed with ViromeXplore. The classification with Kaiju corresponds well with the abundances that were used to generate the synthetic viral community. This was expected given that the viruses used to generate the dataset are well characterized and are present in the database that Kaiju uses by default in ViromeXplore. The results also showed that most viruses were accurately assembled and retrieved with the exception of a giant virus belonging to the mimivirus genus. This result highlights the difficulty in obtaining high-quality and complete NCLDV from metagenomes which has been reported in other comprehensive metagenomic surveys that retrieved mostly medium-quality NCLDV Metagenome-Assembled Genomes (MAGs) [76]. These results and the synthetic viral community benchmark will inform future decisions to incorporate new tools when available, that can further improve viral detection.

## Conclusion

ViromeXplore contains modular and the most comprehensive workflows to explore viral genes and genomes from viromes and metagenomes to date. The workflows in this software are interoperable and can be integrated with other recent workflows for upstream and downstream analysis. We provide two demonstration analyses to illustrate the broad use cases of ViromeXplore; analysis of human virome datasets revealed a broad diversity of viruses and new taxa, and analysis of a food metagenome dataset demonstrated reconstruction of the full genome of a new *Streptococcus* phage that contains genomic rearrangements compared to other related genomes. Furthermore, ViromeXplore was successfully used to analyze a taxonomically diverse synthetic virome. The future aim for ViromeXplore is to continue expanding the workflows and including the most recent tools in order to keep pace with the quickly evolving viromics bioinformatics landscape. Furthermore, continuous integration with the QIIME 2 framework is planned as additional new plugins and integrated tools for metagenome and virome analysis are released, allowing integration between ViromeXplore with a comprehensive platform that enables further analysis and visualization tools. Finally, ViromeXplore supports full virome analysis at multiple levels and from different starting points including raw reads, contigs, genomes, abundance tables, phylogenies, and taxonomies. This will allow flexible and comprehensive analysis of virome datasets, which we hope will support further advances in the field of exploring the sophisticated ecological roles of viromes in diverse global ecosystems.

**Key Points**

- ViromeXplore enables untargeted, automated virome characterization while being compatible and interoperable with other viromics packages and popular bioinformatics platforms.
- The functionality of ViromeXplore is showcased by analyzing human viromes that present a broad taxonomic diversity and a fermented food virome from which a complete genome was obtained.
- ViromeXplore includes new functionality not included in other workflows such as virome contamination estimation, protein-based taxonomic characterization with Kaiju, viral genome contiguity and completeness improvement and network-based host prediction.

## Supplementary Material

Supplementary_file_1_bbaf638

Supplementary_file_2_bbaf638

## Data Availability

The metagenomic sequencing data from the human viromes analyzed in this study are publicly available in the NCBI Sequence Read Archive (SRA) under the following run accessions: SRR7892428, SRR7892429, SRR7892431, SRR7892432, SRR7892433, SRR7892434, SRR7892435, SRR7892437, SRR7892442, SRR7892443, SRR7892458, SRR7892459, SRR7892460, SRR7892461, SRR7892469, SRR7892470, SRR7892477. The metagenomic sequencing data used in this study for the Ryazhenka sample are publicly available in the European Nucleotide Archive (ENA) under accession number ERR4757833.

## Code availability and dependencies

The Nextflow workflows and helper scripts for visualization are available via GitHub: https://github.com/rhernandvel/ViromeXplore

Nextflow version 22.04.4 or later is required.

Databases: All required databases are automatically downloaded by the workflow.

Languages and frameworks: Nextflow, Docker, Python, and Julia helper scripts were also used in the containers.

The documentation and tutorial are available via Read the Docs: https://viromexplore.readthedocs.io

License: BSD 3-Clause License.

## Notes

The authors are researchers at ETH Zurich, the Swiss Federal Institutes of Technology. R.H.-V. is a PhD researcher focusing on food viromics. M.Z. is a senior software engineer. N.A.B. is an assistant professor with broad expertise in microbial bioinformatics, microbial ecology, and microbiomes.

## References

[1] Zeng S, Almeida A, Li S. et al. A metagenomic catalog of the early-life human gut virome. *Nat Commun* 2024;15:1864. 10.1038/s41467-024-45793-z. 38424077 PMC10904392

[2] Ritz NL, Draper LA, Bastiaanssen TFS. et al. The gut virome is associated with stress-induced changes in behaviour and immune responses in mice. *Nat Microbiol* 2024;9:359–76. 10.1038/s41564-023-01564-y. 38316929 PMC10847049

[3] Roux S, Brum JR, Dutilh BE. et al. Ecogenomics and potential biogeochemical impacts of globally abundant ocean viruses. *Nature* 2016;537:689–93. 10.1038/nature19366. 27654921

[4] Vanni C, Schechter MS, Acinas SG. et al. Unifying the known and unknown microbial coding sequence space. *elife* 2022;11:e67667. 10.7554/eLife.67667. 35356891 PMC9132574

[5] Conceição-Neto N, Zeller M, Lefrère H. et al. Modular approach to customise sample preparation procedures for viral metagenomics: A reproducible protocol for virome analysis. *Sci Rep* 2015;5:16532. 10.1038/srep16532. 26559140 PMC4642273

[6] Antipov D, Raiko M, Lapidus A. et al. MetaviralSPAdes: Assembly of viruses from metagenomic data. *Bioinformatics* 2020;36:4126–9. 10.1093/bioinformatics/btaa490. 32413137

[7] Guo J, Bolduc B, Zayed AA. et al. VirSorter2: A multi-classifier, expert-guided approach to detect diverse DNA and RNA viruses. *Microbiome* 2021;9:37. 33522966 10.1186/s40168-020-00990-y PMC7852108

[8] Ren J, Ahlgren NA, Lu YY. et al. VirFinder: A novel k-mer based tool for identifying viral sequences from assembled metagenomic data. *Microbiome* 2017;5:69. 28683828 10.1186/s40168-017-0283-5 PMC5501583

[9] Bin Jang H, Bolduc B, Zablocki O. et al. Taxonomic assignment of uncultivated prokaryotic virus genomes is enabled by gene-sharing networks. *Nat Biotechnol* 2019;37:632–9. 10.1038/s41587-019-0100-8. 31061483

[10] Marquet M, Hölzer M, Pletz MW. et al. What the phage: A scalable workflow for the identification and analysis of phage sequences. *Gigascience* 2022;11:giac110. 10.1093/gigascience/giac110 PMC9673492 36399058

[11] Rangel-Pineros G, Almeida A, Beracochea M. et al. VIRify: An integrated detection, annotation and taxonomic classification pipeline using virus-specific protein profile hidden Markov models. *PLoS Comput Biol* 2023;19:e1011422. 10.1371/journal.pcbi.1011422. 37639475 PMC10491390

[12] Wang X, Ding Z, Yang Y. et al. ViromeFlowX: A comprehensive Nextflow-based automated workflow for mining viral genomes from metagenomic sequencing data. *Microb Genom* 2024;10:10. 10.1099/mgen.0.001202. PMC10926697 38381034

[13] Di Tommaso P, Chatzou M, Floden EW. et al. Nextflow enables reproducible computational workflows. *Nat Biotechnol* 2017;35:316–9. 10.1038/nbt.3820. 28398311

[14] Coclet C, Camargo AP, Roux S. MVP: A modular viromics pipeline to identify, filter, cluster, annotate, and bin viruses from metagenomes. *mSystems* 2024;9:e00888-24. 10.1128/msystems.00888-24 PMC11498083 39352141

[15] Li Y, Wang H, Nie K. et al. VIP: An integrated pipeline for metagenomics of virus identification and discovery. *Sci Rep* 2016;6:23774. 10.1038/srep23774. 27026381 PMC4824449

[16] Fuhrmann L, Jablonski KP, Topolsky I. et al. V-pipe 3.0: A sustainable pipeline for within-sample viral genetic diversity estimation. *Gigascience* 2024;13:giae065. 10.1093/gigascience/giae065 PMC11440432 39347649

[17] Pratas D, Toppinen M, Pyöriä L. et al. A hybrid pipeline for reconstruction and analysis of viral genomes at multi-organ level. *Gigascience* 2020;9:giaa086. 10.1093/gigascience/giaa086 PMC7439602 32815536

[18] Bolyen E, Rideout JR, Dillon MR. et al. Reproducible, interactive, scalable and extensible microbiome data science using QIIME 2. *Nat Biotechnol* 2019;37:852–7. 10.1038/s41587-019-0209-9. 31341288 PMC7015180

[19] Keefe CR, Dillon MR, Gehret E. et al. Facilitating bioinformatics reproducibility with QIIME 2 provenance replay. *PLoS Comput Biol* 2023;19:e1011676. 10.1371/journal.pcbi.1011676. 38011287 PMC10703398

[20] Ziemski M, Gehret L, Simard A., et al. MOSHPIT: Accessible, Reproducible Metagenome Data Science on the QIIME 2 Framework. 2025. arXiv. 10.1101/2025.01.27.635007;42785295

[21] Zolfo M, Pinto F, Asnicar F. et al. Detecting contamination in viromes using ViromeQC. *Nat Biotechnol* 2019;37:1408–12. 10.1038/s41587-019-0334-5. 31748692

[22] Camargo AP, Roux S, Schulz F. et al. Identification of mobile genetic elements with geNomad. *Nat Biotechnol* 2024;42:1303–12. 10.1038/s41587-023-01953-y. 37735266 PMC11324519

[23] Mihara T, Nishimura Y, Shimizu Y. et al. Linking virus genomes with host taxonomy. *Viruses* 2016;8:8. 10.3390/v8030066. PMC4810256 26938550

[24] Menzel P, Ng KL, Krogh A. Fast and sensitive taxonomic classification for metagenomics with Kaiju. *Nat Commun* 2016;7:11257. 27071849 10.1038/ncomms11257 PMC4833860

[25] Ondov BD, Bergman NH, Phillippy AM. Interactive metagenomic visualization in a web browser. *BMC Bioinformatics* 2011;12:385. 21961884 10.1186/1471-2105-12-385 PMC3190407

[26] Chen S, Zhou Y, Chen Y. et al. Fastp: An ultra-fast all-in-one FASTQ preprocessor. *Bioinformatics* 2018;34:i884–90. 10.1093/bioinformatics/bty560. 30423086 PMC6129281

[27] Li D, Liu C-M, Luo R. et al. MEGAHIT: An ultra-fast single-node solution for large and complex metagenomics assembly via succinct de Bruijn graph. *Bioinformatics* 2015;31:1674–6. 10.1093/bioinformatics/btv033. 25609793

[28] Nayfach S, Camargo AP, Schulz F. et al. CheckV assesses the quality and completeness of metagenome-assembled viral genomes. *Nat Biotechnol* 2021;39:578–85. 10.1038/s41587-020-00774-7. 33349699 PMC8116208

[29] Li W, Godzik A. Cd-hit: A fast program for clustering and comparing large sets of protein or nucleotide sequences. *Bioinformatics* 2006;22:1658–9. 10.1093/bioinformatics/btl158. 16731699

[30] Wu Z, Liu T, Chen Q. et al. Unveiling the unknown viral world in groundwater. *Nat Commun* 2024;15:6788. 39117653 10.1038/s41467-024-51230-y PMC11310336

[31] Yan M, Pratama AA, Somasundaram S. et al. Interrogating the viral dark matter of the rumen ecosystem with a global virome database. *Nat Commun* 2023;14:5254. 37644066 10.1038/s41467-023-41075-2 PMC10465536

[32] Gregory AC, Zayed AA, Conceição-Neto N. et al. Marine DNA viral macro- and microdiversity from pole to pole. *Cell* 2019;177:1109–1123.e14. 10.1016/j.cell.2019.03.040. 31031001 PMC6525058

[33] Rognes T, Flouri T, Nichols B. et al. VSEARCH: A versatile open source tool for metagenomics. *PeerJ* 2016;4:e2584. 10.7717/peerj.2584. 27781170 PMC5075697

[34] Cantalapiedra CP, Hernández-Plaza A, Letunic I. et al. eggNOG-mapper v2: Functional annotation, Orthology assignments, and domain prediction at the metagenomic scale. *Mol Biol Evol* 2021;38:5825–9. 10.1093/molbev/msab293. 34597405 PMC8662613

[35] Buchfink B, Xie C, Huson DH. Fast and sensitive protein alignment using DIAMOND. *Nat Methods* 2015;12:59–60. 10.1038/nmeth.3176. 25402007

[36] Huerta-Cepas J, Szklarczyk D, Heller D. et al. eggNOG 5.0: A hierarchical, functionally and phylogenetically annotated orthology resource based on 5090 organisms and 2502 viruses. *Nucleic Acids Res* 2019;47:D309–14. 10.1093/nar/gky1085. 30418610 PMC6324079

[37] Langmead B, Salzberg SL. Fast gapped-read alignment with bowtie 2. *Nat Methods* 2012;9:357–9. 10.1038/nmeth.1923. 22388286 PMC3322381

[38] Danecek P, Bonfield JK, Liddle J. et al. Twelve years of SAMtools and BCFtools. *Gigascience* 2021;10:giab008. 33590861 10.1093/gigascience/giab008 PMC7931819

[39] Kang DD, Li F, Kirton E. et al. MetaBAT 2: An adaptive binning algorithm for robust and efficient genome reconstruction from metagenome assemblies. *PeerJ* 2019;7:e7359. 10.7717/peerj.7359. 31388474 PMC6662567

[40] Chen L, Banfield JF. COBRA improves the completeness and contiguity of viral genomes assembled from metagenomes. *Nat Microbiol* 2024;9:737–50. 10.1038/s41564-023-01598-2. 38321183 PMC10914622

[41] Kaneko H, Blanc-Mathieu R, Endo H. et al. Eukaryotic virus composition can predict the efficiency of carbon export in the global ocean. *iScience* 2021;24:102002. 10.1016/j.isci.2020.102002. PMC7811142 33490910

[42] Tackmann J, Matias Rodrigues JF, von Mering C. Rapid inference of direct interactions in large-scale ecological networks from heterogeneous microbial sequencing data. *Cell Syst* 2019;9:286–296.e8. 10.1016/j.cels.2019.08.002. 31542415

[43] Letunic I, Bork P. Interactive tree of life (iTOL) v6: Recent updates to the phylogenetic tree display and annotation tool. *Nucleic Acids Res* 2024;52:W78–82. 10.1093/nar/gkae268. 38613393 PMC11223838

[44] Yinda CK, Vanhulle E, Conceição-Neto N. et al. Gut Virome analysis of Cameroonians reveals high diversity of enteric viruses, including potential interspecies transmitted viruses. *mSphere* 2019;4:10.1128/msphere.00585-18. 10.1128/msphere.00585-18. PMC6344602 30674646

[45] Ziemski M, Adamov A, Kim L. et al. Reproducible acquisition, management and meta-analysis of nucleotide sequence (meta)data using q2-fondue. *Bioinformatics* 2022;38:5081–91. 10.1093/bioinformatics/btac639. 36130056 PMC9665871

[46] Camacho C, Coulouris G, Avagyan V. et al. BLAST+: Architecture and applications. *BMC Bioinformatics* 2009;10:421. 20003500 10.1186/1471-2105-10-421 PMC2803857

[47] Seemann T . Prokka: Rapid prokaryotic genome annotation. *Bioinformatics* 2014;30:2068–9. 10.1093/bioinformatics/btu153. 24642063

[48] Huang W, Li L, Myers JR. et al. ART: A next-generation sequencing read simulator. *Bioinformatics* 2012;28:593–4. 10.1093/bioinformatics/btr708. 22199392 PMC3278762

[49] Mikheenko A, Saveliev V, Gurevich A. MetaQUAST: Evaluation of metagenome assemblies. *Bioinformatics* 2016;32:1088–90. 10.1093/bioinformatics/btv697. 26614127

[50] Ye SH, Siddle KJ, Park DJ. et al. Benchmarking metagenomics tools for taxonomic classification. *Cell* 2019;178:779–94. 10.1016/j.cell.2019.07.010. 31398336 PMC6716367

[51] Zhang Z, Yang C, Veldsman WP. et al. Benchmarking genome assembly methods on metagenomic sequencing data. *Brief Bioinform* 2023;24:bbad087. 36917471 10.1093/bib/bbad087

[52] Zayed AA, Wainaina JM, Dominguez-Huerta G. et al. Cryptic and abundant marine viruses at the evolutionary origins of Earth’s RNA virome. *Science* 1979;2022:156–62. 10.1126/science.abm5847 PMC10990476 35389782

[53] Sun J, Qiu Z, Egan R. et al. Persistent memory as an effective alternative to random access memory in metagenome assembly. *BMC Bioinformatics* 2022;23:513. 36451083 10.1186/s12859-022-05052-8 PMC9710083

[54] Ho SFS, Wheeler NE, Millard AD. et al. Gauge your phage: Benchmarking of bacteriophage identification tools in metagenomic sequencing data. *Microbiome* 2023;11:84. 37085924 10.1186/s40168-023-01533-x PMC10120246

[55] Amgarten D, Braga LPP, da Silva AM. et al. MARVEL, a tool for prediction of bacteriophage sequences in metagenomic bins. *Front Genet* 2018;9:304. 10.3389/fgene.2018.00304. PMC6090037 30131825

[56] Jain C, Rodriguez-R LM, Phillippy AM. et al. High throughput ANI analysis of 90K prokaryotic genomes reveals clear species boundaries. *Nat Commun* 2018;9:5114. 30504855 10.1038/s41467-018-07641-9 PMC6269478

[57] Eren AM. Comparing different mapping software using anvi'o. https://merenlab.org/2015/06/23/comparing-different-mapping-software/ (21 November 2025, date last accessed).

[58] Pasolli E, Asnicar F, Manara S. et al. Extensive unexplored human microbiome diversity revealed by over 150,000 genomes from metagenomes spanning age, geography, and lifestyle. *Cell* 2019;176:649–662.e20. 10.1016/j.cell.2019.01.001. 30661755 PMC6349461

[59] Huerta-Cepas J, Forslund K, Coelho LP. et al. Fast genome-wide functional annotation through Orthology assignment by eggNOG-mapper. *Mol Biol Evol* 2017;34:2115–22. 10.1093/molbev/msx148. 28460117 PMC5850834

[60] Wood DE, Lu J, Langmead B. Improved metagenomic analysis with Kraken 2. *Genome Biol* 2019;20:257. 31779668 10.1186/s13059-019-1891-0 PMC6883579

[61] Bolger AM, Lohse M, Usadel B. Trimmomatic: A flexible trimmer for Illumina sequence data. *Bioinformatics* 2014;30:2114–20. 10.1093/bioinformatics/btu170. 24695404 PMC4103590

[62] Nurk S, Meleshko D, Korobeynikov A. et al. metaSPAdes: A new versatile metagenomic assembler. *Genome Res* 2017;27:824–34. 10.1101/gr.213959.116. 28298430 PMC5411777

[63] Fang Z, Tan J, Wu S. et al. PPR-meta: A tool for identifying phages and plasmids from metagenomic fragments using deep learning. *Gigascience* 2019;8:giz066. 10.1093/gigascience/giz066. PMC6586199 31220250

[64] Aroney STN, Newell RJP, Nissen JN. et al. CoverM: Read alignment statistics for metagenomics. *Bioinformatics* 2025;41:btaf147. 10.1093/bioinformatics/btaf147. PMC11993303 40193404

[65] Quinlan AR, Hall IM. BEDTools: A flexible suite of utilities for comparing genomic features. *Bioinformatics* 2010;26:841–2. 10.1093/bioinformatics/btq033. 20110278 PMC2832824

[66] Potter SC, Luciani A, Eddy SR. et al. HMMER web server: 2018 update. *Nucleic Acids Res* 2018;46:W200–4. 29905871 10.1093/nar/gky448 PMC6030962

[67] Li W, O’Neill KR, Haft DH. et al. RefSeq: Expanding the prokaryotic genome annotation pipeline reach with protein family model curation. *Nucleic Acids Res* 2021;49:D1020–8. 10.1093/nar/gkaa1105. 33270901 PMC7779008

[68] Finn RD, Coggill P, Eberhardt RY. et al. The Pfam protein families database: Towards a more sustainable future. *Nucleic Acids Res* 2016;44:D279–85. 10.1093/nar/gkv1344. 26673716 PMC4702930

[69] Ryan F. Demovir. https://github.com/feargalr/Demovir (21 November 2025, date last accessed).

[70] Suzek BE, Wang Y, Huang H. et al. UniRef clusters: A comprehensive and scalable alternative for improving sequence similarity searches. *Bioinformatics* 2015;31:926–32. 10.1093/bioinformatics/btu739. 25398609 PMC4375400

[71] Andino R, Kirkegaard K, Macadam A. et al. The Picornaviridae family: Knowledge gaps, animal models, countermeasures, and prototype pathogens. *J Infect Dis* 2023;228:S427–45. 10.1093/infdis/jiac426. 37849401 PMC13381290

[72] Leech J, Cabrera-Rubio R, Walsh AM. et al. Fermented-food metagenomics reveals substrate-associated differences in taxonomy and health-associated and antibiotic resistance determinants. *mSystems* 2020;5:10.1128/msystems.00522-20. 10.1128/mSystems.00522-20. PMC7657593 33172966

[73] Mills S, Griffin C, O’Sullivan O. et al. A new phage on the ‘mozzarella’ block: Bacteriophage 5093 shares a low level of homology with other Streptococcus thermophilus phages. *Int Dairy J* 2011;21:963–9. 10.1016/j.idairyj.2011.06.003.

[74] Lefkowitz EJ, Dempsey DM, Hendrickson RC. et al. Virus taxonomy: The database of the International Committee on Taxonomy of Viruses (ICTV). *Nucleic Acids Res* 2018;46:D708–17. 10.1093/nar/gkx932. 29040670 PMC5753373

[75] Blanco-Picazo P, Gómez-Gómez C, Tormo M. et al. Prevalence of bacterial genes in the phage fraction of food viromes. *Food Res Int* 2022;156:111342. 10.1016/j.foodres.2022.111342. 35651089

[76] Schulz F, Roux S, Paez-Espino D. et al. Giant virus diversity and host interactions through global metagenomics. *Nature* 2020;578:432–6. 10.1038/s41586-020-1957-x. 31968354 PMC7162819

