# Supplementary material for: ViromeXplore: integrative workflows for complete and reproducible virome characterization
Source: Brief Bioinform. 2025 Dec 5;26(6):bbaf638. doi: 10.1093/bib/bbaf638 (PMC12862488; doi:10.1093/bib/bbaf638)
Supplement: Supplementary_file_1_bbaf638 [file supplementary_file_1_bbaf638.docx]

| Virus | NCBI Definition | NCBI type | Genome_size | NCBI_accession |
| --- | --- | --- | --- | --- |
| Severe acute respiratory syndrome coronavirus 2 (SARS-CoV-2) | Severe acute respiratory syndrome coronavirus 2 isolate Wuhan-Hu-1, complete genome | ss-RNA linear | 29,903 bp | NC_045512.2 |
| Human betacoronavirus 2c EMC/2012 | Human betacoronavirus 2c EMC/2012, complete genome | RNA linear | 30,119 bp | JX869059.2 |
| Zaire ebolavirus | Zaire ebolavirus strain Ebola_virus/DRC/Likati/Human/2017/1, complete genome | cRNA linear | 18,898 bp | MH481611 |
| Zika virus | Zika virus isolate Zika virus/H.sapiens-tc/KHM/2010/FSS13025, complete genome | ss-RNA linear | 10,807 bp | KU955593.1 |
| Human respiratory syncytial virus (HRSV) | Human respiratory syncytial virus wildtype strain B1, complete genome | RNA linear | 15,225 bp | AF013254.1 |
| Acanthamoeba polyphaga mimivirus | Acanthamoeba polyphaga mimivirus, complete genome | DNA linear | 1,181,549 bp | NC_014649.1 |
| Escherichia phage T4 | Enterobacteria phage T4, complete genome | DNA linear | 168,903 bp | NC_000866.4 |
| Escherichia phage Lambda | Enterobacteria phage lambda, complete genome | DNA linear | 48,502 bp | NC_001416.1 |
| Escherichia phage phiX174 | Escherichia phage phiX174, complete genome. | ss-DNA circular | 5,386 bp | NC_001422.1 |
| Bacteroides phage crAss001 | Bacteroides phage crAss001, complete genome. | DNA circular | 102,679 bp | NC_049977.1 |
| Escherichia phage MS2 | phage MS2 genome | RNA linear | 3,569 bp | NC_001417.2 |
| Saccharomyces cerevisiae virus L-A | Saccharomyces cerevisiae virus L-A (L1), complete genome | ds-RNA linear | 4,579 bp | NC_003745.1 |
| Sulfolobus turreted icosahedral virus 1 | Sulfolobus turreted icosahedral virus, complete genome | DNA circular | 17,663 bp | NC_005892.1 |
| Prochlorococcus phage P-SSM2 | Prochlorococcus phage P-SSM2, complete genome | DNA linear | 252,401 bp | NC_006883.2 |
| Salmonella phage P22 | Salmonella phage P22, complete genome. | DNA linear | 41,724 bp | NC_002371.2 |

Supplementary file 1: Viral genomes used to generate the synthetic viral community.
