# Supplementary material for: ViromeXplore: integrative workflows for complete and reproducible virome characterization
Source: Brief Bioinform. 2025 Dec 5;26(6):bbaf638. doi: 10.1093/bib/bbaf638 (PMC12862488; doi:10.1093/bib/bbaf638)
Supplement: Supplementary_file_2_bbaf638 [file supplementary_file_2_bbaf638.docx]

| genome_id | proportion_of_reads | genome_length | read_pairs | coverage |
| --- | --- | --- | --- | --- |
| NC_014649.1 | 0.26114581 | 1181549 | 5222916 | 1326.119238 |
| MH481611.2 | 0.216469769 | 18898 | 4329395 | 68727.83437 |
| NC_001417.2 | 0.144879025 | 3569 | 2897580 | 243562.3844 |
| NC_000866.4 | 0.05798013 | 168903 | 1159603 | 2059.648319 |
| NC_005892.1 | 0.053942932 | 17663 | 1078859 | 18324.04416 |
| NC_003745.1 | 0.051355781 | 4579 | 1027116 | 67293.00852 |
| AF013254.1 | 0.038944837 | 15225 | 778897 | 15347.71901 |
| NC_006883.2 | 0.038084988 | 252401 | 761700 | 905.3447807 |
| KU955593.1 | 0.036292496 | 10807 | 725850 | 20149.43796 |
| JX869059.2 | 0.025299584 | 30119 | 505992 | 5039.925097 |
| NC_049977.1 | 0.019534977 | 102679 | 390700 | 1141.51737 |
| NC_002371.2 | 0.017115621 | 41724 | 342312 | 2461.262726 |
| NC_045512.2 | 0.017020698 | 29903 | 340414 | 3415.182022 |
| NC_001422.1 | 0.01462566 | 5386 | 292513 | 16292.97438 |
| NC_001416.1 | 0.007307692 | 48502 | 146154 | 904.0070925 |

Supplementary file 2: Table containing the number of reads used for generating the synthetic virome and their corresponding proportion of the total reads.
